# Supplementary material for: Large language models streamline automated machine learning for clinical studies
Source: Nat Commun. 2024 Feb 21;15:1603. doi: 10.1038/s41467-024-45879-8 (PMC10881983; doi:10.1038/s41467-024-45879-8)
Supplement: Supplementary file 2 — Reporting Summary [file 41467_2024_45879_MOESM2_ESM.pdf]

nature portfolio

Corresponding author(s): STA, TH

Last updated by author(s): Dec 22, 2023

Reporting Summary

Nature Portfolio wishes to improve the reproducibility of the work that we publish. This form provides structure for consistency and transparency in reporting. For further information on Nature Portfolio policies, see our Editorial Policies and the Editorial Policy Checklist.

Statistics

For all statistical analyses, confirm that the following items are present in the figure legend, table legend, main text, or Methods section.

n/a	Confirmed	
☐	☒	The exact sample size (*n*) for each experimental group/condition, given as a discrete number and unit of measurement
☐	☒	A statement on whether measurements were taken from distinct samples or whether the same sample was measured repeatedly
☐	☒	The statistical test(s) used AND whether they are one- or two-sided<br>*Only common tests should be described solely by name; describe more complex techniques in the Methods section.*
☐	☒	A description of all covariates tested
☐	☒	A description of any assumptions or corrections, such as tests of normality and adjustment for multiple comparisons
☐	☒	A full description of the statistical parameters including central tendency (e.g. means) or other basic estimates (e.g. regression coefficient) AND variation (e.g. standard deviation) or associated estimates of uncertainty (e.g. confidence intervals)
☐	☒	For null hypothesis testing, the test statistic (e.g. *F*, *t*, *r*) with confidence intervals, effect sizes, degrees of freedom and *P* value noted<br>*Give P values as exact values whenever suitable.*
☒	☐	For Bayesian analysis, information on the choice of priors and Markov chain Monte Carlo settings
☒	☐	For hierarchical and complex designs, identification of the appropriate level for tests and full reporting of outcomes
☒	☐	Estimates of effect sizes (e.g. Cohen's *d*, Pearson's *r*), indicating how they were calculated

Our web collection on statistics for biologists contains articles on many of the points above.

Software and code

Policy information about availability of computer code

Data collection	No software was used for data collection.
Data analysis	ChatGPT Advanced Data Analysis (previously known as "Code Interpreter") was utilized for analyses and may be accessed via https://chat.openai.com for ChatGPT Plus users. Source codes for training, evaluating, and optimizing the ML models, as well as for data pre-processing, statistical analysis, and visualizations, are publicly available at https://github.com/tayebiarasteh/LLMmed. The code was developed in Python v3.9.18 using open-source libraries including shap (v0.44.0), numpy (v1.26.2), scipy (v1.11.4), lightgbm (v4.1.0), mne (v1.6.0), pandas (v2.1.1), and scikit-learn (v1.3.0).

For manuscripts utilizing custom algorithms or software that are central to the research but not yet described in published literature, software must be made available to editors and reviewers. We strongly encourage code deposition in a community repository (e.g. GitHub). See the Nature Portfolio guidelines for submitting code & software for further information.

Data

Policy information about availability of data

All manuscripts must include a data availability statement. This statement should provide the following information, where applicable:

- Accession codes, unique identifiers, or web links for publicly available datasets
- A description of any restrictions on data availability
- For clinical datasets or third party data, please ensure that the statement adheres to our policy

The datasets utilized in this study were extracted from public repositories. The raw data for predicting metastatic disease in pheochromocytoma or paraganglioma is

available on Zenodo: https://doi.org/10.5281/zenodo.7749613. The Oesophageal cancer screening trial data is available on GitHub: https://github.com/Gaooooye/Esophageal-cAncer-Screening-Trial. The hereditary hearing loss trial data is available on Mendeley: https://data.mendeley.com/datasets/6mh8mpnbgv/1. The data on cardiac amyloidosis ("derivation dataset") is available per the original study at https://www.nature.com/articles/s41467-021-22876-9.

Research involving human participants, their data, or biological material

Policy information about studies with human participants or human data. See also policy information about sex, gender (identity/presentation), and sexual orientation and race, ethnicity and racism.

Reporting on sex and gender	Not applicable as the data used in this study was collected from previously published clinical trials for the subsequent data analysis.
Reporting on race, ethnicity, or other socially relevant groupings	Not applicable as the data used in this study was collected from previously published clinical trials for the subsequent data analysis.
Population characteristics	The data used in this study was collected from previously published clinical trials for the subsequent data analysis.<br><br>1) Endocrinologic Oncology data: Total subjects included 493 as training and 295 test subjects. Training subjects had an age range of 4 to 83 with a mean of 42 ± 18 (Mean ± Standard Deviation) and a median of 42 years. Test subjects had an age range of 11 to 82 with a mean of 47 ± 16 (Mean ± Standard Deviation) and a median of 48 years.<br><br>2) Gastrointestinal Oncology data: Total subjects included 7899 as training and 6698 test subjects. Training subjects had an age range of 39 to 82 with a mean of 56 ± 9 (Mean ± Standard Deviation) and a median of 56 years. Test subjects had an age range of 24 to 86 with a mean of 56 ± 9 (Mean ± Standard Deviation) and a median of 55 years.<br><br>3) Otolaryngology data: Total subjects included 1329 as training and 569 test subjects. Training subjects had a mean age of 18 ± 15 (Mean ± Standard Deviation). Test subjects had a mean age of 34 ± 12 (Mean ± Standard Deviation).<br><br>4) Cardiology data: Total subjects included 1712 as training and 430 test subjects. In this study, the original data split and, consequently, the external validation dataset was unavailable per the original study. In line with the published methodology, we randomly allocated 80% of patients and controls to the training set (n=1712) and 20% to the test set (n=430).
Recruitment	Not applicable as the data used in this study was collected from previously published clinical trials for the subsequent data analysis.
Ethics oversight	The study followed relevant guidelines and regulations and was approved by the Ethical Committee of the Medical Faculty of RWTH Aachen University (Reference No. EK 028/19).

Note that full information on the approval of the study protocol must also be provided in the manuscript.

Field-specific reporting

Please select the one below that is the best fit for your research. If you are not sure, read the appropriate sections before making your selection.

[x] Life sciences [ ] Behavioural & social sciences [ ] Ecological, evolutionary & environmental sciences

For a reference copy of the document with all sections, see nature.com/documents/nr-reporting-summary-flat.pdf

Life sciences study design

All studies must disclose on these points even when the disclosure is negative.

Sample size	We extracted the publicly available original training and test datasets from each clinical trial.<br>1) Endocrinologic Oncology data: Total subjects included 493 as training and 295 test subjects. Cross-sectional data from Germany, Poland, the US, and the Netherlands was used to assess the ability of the dopamine metabolite methoxytyramine to identify metastatic disease in patients with pheochromocytoma or paraganglioma. To this end, ten features were available.<br><br>2) Gastrointestinal Oncology data: Total subjects included 7899 as training and 6698 test subjects. It was centered on endoscopic screening and included multiple data sources from questionnaires to endoscopy data, i.e., cytologic and epidemiologic data.<br><br>3) Otolaryngology data: Total subjects included 1329 as training and 569 test subjects. The study contained genetic sequencing data to diagnose this condition. Individuals were categorized based on hearing loss severity and variations in three genes (GJB2, SLC26A4, MT-RNR1).<br><br>4) Cardiology data: Total subjects included 1712 as training and 430 test subjects. The study utilized electronic health records to identify patients with cardiac amyloidosis from a dataset spanning 2008-2019, sourced from IQVIA, Inc., focusing on heart failure and amyloidosis. While the original study used external datasets for validation, these were inaccessible. Therefore, our analysis adhered to the original study's internal validation strategy: 80% as the training set and 20% for testing, resulting in 1712 individuals for training and 430 for testing.
Data exclusions	No publicly available data was excluded. The data used in this study was collected from previously published clinical trials for the subsequent data analysis.

Replication	Not applicable as the data used in this study was collected from previously published clinical trials for the subsequent data analysis.
Randomization	Randomization was not applicable to this study as the data used in this study was collected from previously published clinical trials for the subsequent data analysis. For Endocrinologic Oncology data, Gastrointestinal Oncology data, and Otolaryngology data, the same data subsets as the original studies was utilized.
Blinding	Blinding was not applicable to this study as the data used in this study was collected from previously published clinical trials for the subsequent data analysis. For Cardiology data, the same training data set as the original study was utilized. However, as the external validation data were not publicly available, only the internal validation was utilized. Therefore, our analysis adhered to the original study's internal validation strategy: 80% as the training set and 20% for testing, resulting in 1712 individuals for training and 430 for testing. This division was performed randomly.

Reporting for specific materials, systems and methods

We require information from authors about some types of materials, experimental systems and methods used in many studies. Here, indicate whether each material, system or method listed is relevant to your study. If you are not sure if a list item applies to your research, read the appropriate section before selecting a response.

Materials & experimental systems

n/a	Involved in the study
☒	☐ Antibodies
☒	☐ Eukaryotic cell lines
☒	☐ Palaeontology and archaeology
☒	☐ Animals and other organisms
☐	☒ Clinical data
☒	☐ Dual use research of concern
☒	☐ Plants

Methods

n/a	Involved in the study
☒	☐ ChIP-seq
☒	☐ Flow cytometry
☒	☐ MRI-based neuroimaging

Clinical data

Policy information about clinical studies

All manuscripts should comply with the ICMJEguidelines for publication of clinical research and a completedCONSORT checklist must be included with all submissions.

Clinical trial registration	Not applicable as the data used in this study was collected from previously published clinical trials for the subsequent data analysis.
Study protocol	Not applicable as the data used in this study was collected from previously published clinical trials for the subsequent data analysis.
Data collection	Not applicable as the data used in this study was collected from previously published clinical trials for the subsequent data analysis.
Outcomes	Not applicable as the data used in this study was collected from previously published clinical trials for the subsequent data analysis.

Plants

Seed stocks	Not Applicable.
Novel plant genotypes	Not Applicable.
Authentication	Not Applicable.
